# Transforming growth factor beta 1 levels predict echocardiographic changes at three years after adjuvant radiotherapy for breast cancer

**DOI:** 10.1186/s13014-019-1366-1

**Published:** 2019-08-30

**Authors:** Hanna Aula, Tanja Skyttä, Suvi Tuohinen, Tiina Luukkaala, Mari Hämäläinen, Vesa Virtanen, Pekka Raatikainen, Eeva Moilanen, Pirkko-Liisa Kellokumpu-Lehtinen

**Affiliations:** 1 Faculty of Medicine and Health Technology, Tampere University, 33014 Tampere, Finland; 2 Department of Oncology, Tampere University Hospital, PO Box 2000, 33521 Tampere, Finland; 3 Heart Hospital, Tampere University Hospital, PO Box 2000, 33521 Tampere, Finland; 4 Department of Cardiology, Heart and Lung Center, Helsinki University Hospital, PO Box 340, 00029 HUS Helsinki, Finland; 5 Research, Innovation and Development Center, Tampere University Hospital, PO Box 2000, 33521 Tampere, Finland; 6 Health Sciences, Faculty of Social Sciences, Tampere University, 33014 Tampere, Finland; 7 The Immunopharmacology Research Group, Faculty of Medicine and Health Technology, Tampere University and Tampere University Hospital, 33014 Tampere, Finland

**Keywords:** Transforming growth factor beta 1, Platelet-derived growth factor, Cardiotoxicity, Breast cancer, Radiotherapy, Echocardiography

## Abstract

**Background:**

Transforming growth factor beta 1 (TGF-β1) and platelet-derived growth factor (PDGF) are cytokines involved in fibrotic processes causing radiotherapy (RT)-induced cardiovascular changes. We aimed to investigate the associations between TGF-β1 and PDGF and the echocardiographic changes that occur during RT and during three-year follow-up.

**Methods:**

The study included 63 women receiving adjuvant RT for early-stage breast cancer or ductal carcinoma in situ. Serum TGF-β1 (ng/ml) and PDGF (ng/ml) levels were measured by enzyme-linked immunoassay and echocardiographic examination was performed before RT, after RT and at 3 years. Patients were grouped by biomarker behavior by a trajectory analysis.

**Results:**

TGF-β1 decreased from 19.2 (IQR 17.1–22.3) before RT to 18.8 (14.5–22.0) after RT (*p* = 0.003) and the decrease persisted at 17.2 (13.7–21.2) 3 years after RT (*p* = 0.101). PDGF decreased from 15.4 (12.6–19.1) before RT to 13.8 (11.7–16.2) after RT, *p* = 0.001, and persisted at 15.6 (10.4–18.4) at 3 years, *p* = 0.661. The TGF-β1 level before RT (Spearman’s rho 0.441, *p* < 0.001) and the three-year change in TGF-β1 (rho = − 0.302, *p* = 0.018) correlated with global longitudinal strain (GLS) in echocardiography at 3 years.

In trajectory analysis, two TGF-β1 behavior groups were found. Group 1 had significantly higher TGF-β1 levels before RT, 25.6 (22.3–28.6), than group 2, 17.8 (15.9–19.9), *p* < 0.001. In multivariable analysis, TGF-β1 trajectory group 1 (β = 0.27, *p* = 0.013), left-sided breast cancer (β = 0.39, *p* = 0.001) and the use of aromatase inhibitors (β = 0.29, *p* = 0.011) were significantly associated with a worsening in GLS from before RT to 3 years.

**Conclusion:**

An elevated pretreatment TGF-β1 may predict RT-associated changes in echocardiography.

**Electronic supplementary material:**

The online version of this article (10.1186/s13014-019-1366-1) contains supplementary material, which is available to authorized users.

## Background

Adjuvant radiotherapy (RT) of breast cancer is associated with an increased risk of cardiovascular morbidity and mortality [1]. Transforming growth factor beta 1 (TGF-β1) is involved in the process responsible for the long-term fibrotic effects of RT, including cardiovascular changes and morbidity [2]. TGF-β1 is produced by platelets, macrophages, fibroblasts, monocytes, and epithelial and endothelial cells [2]. In a radiation fibrosis model, the production of profibrotic cytokines, such as TGF-β1 and platelet-derived growth factor (PDGF), is thought to be induced by radiation, which in turn activates fibroblasts. It is suggested that cytokines play an initiative role, but may not be necessary to maintain the fibrotic process [2].

TGF-β1 is also released following myocardial injury, exerting profibrotic actions on the myocardium. This process is involved in the pathogenesis of different myocardial diseases [3]. Elevated levels of TGF-β1 are also present in various fibrotic diseases, such as hepatic fibrosis, idiopathic pulmonary fibrosis, myelofibrosis or systemic sclerosis [4]. In addition, variation in circulating TGF-β1 levels is also caused by genetic factors [5]. Mutations in genes determining TGF-β1 levels may be responsible for susceptibility to radiation injury, such as breast fibrosis or increased cardiovascular morbidity [6, 7].

Only a few studies have examined the effect of adjuvant breast cancer RT on the circulating levels of TGF-β1. Two studies have reported that patients who developed fibrosis of the breast as sequelae of external beam RT had higher baseline levels of TGF-β1 than those who did not develop fibrosis [8, 9]. We reported that patients receiving adjuvant RT for breast cancer with echocardiographic changes from before to after RT had higher baseline TGF- β1 levels and their TGF-β1 and PDGF levels decreased during RT [10].

Our aim was to study the behavior of the TGF-β1 and PDGF levels and their association with the echocardiographic changes from before RT to immediately after and 3 years after RT.

## Materials and methods

### Patients

Altogether, 63 patients with available serum samples, receiving postoperative RT for breast cancer or ductal carcinoma in situ (DCIS) were included in this prospective, observational, single-center study. The included patients had breast-conserving surgery (*n* = 62) or a mastectomy (*n* = 1) before RT, but none received chemotherapy. Earlier publication describes the key inclusion and exclusion criteria in detail [11]. The Tampere University hospital ethics committee approved the study (R10160) and informed consent was obtained from all participants.

### Radiotherapy

A detailed description of the RT protocol is found in an earlier publication [12]. Briefly, the planning target volume (PTV) received either 50 Gy in 2 Gy fractions or 42.56 Gy in 2.66 Gy fractions. The remaining breast after breast-conserving surgery or the chest wall after mastectomy with margins constituted the PTV. For the one patient with axillary node-positive disease, PTV included the axillary and supraclavicular areas.

### Serum biomarker analysis

TGF-β1, PDGF and N-terminal pro-brain natriuretic peptide (proBNP) were analyzed from serum samples drawn at the start of RT, at the end of RT and 3 years after RT. Concentrations of TGF-β1 and PDGF-AB were measured by enzyme-linked immunosorbent assay with reagents from R&D Systems Europe Ltd. (Abingdon, UK). Samples were stored at − 80 °C and all samples were analyzed simultaneously with reagents from the same batch. Samples from before and after RT were also reanalyzed [10]. The detection limit and the interassay coefficient of variation were 7.8 ng/ml and 5.1% for TGF-β1 and 3.9 ng/ml and 3.5% for PDGF-AB, respectively.

### Echocardiographic examination

Echocardiographic examination was performed at the same time points as when the serum samples were drawn. A single cardiologist (ST) performed all the examinations by a commercially available ultrasound machine (Philips iE33 ultrasound system; Philips, Bothell, WA, USA) and a 1–5 MHz matrix-array X5–1 transducer, as described previously [13, 14].

### Statistical analysis

The median and interquartile range (IQR) were calculated for variables with skewed distributions. The Wilcoxon signed-rank test was used to test for change in a variable between measurements before RT, after RT and at 3 years. The linear relationship between continuous variables was determined using Spearman’s correlation. The Mann-Whitney U-test for continuous variables was used to test for differences in the biomarkers, echocardiographic measurements or radiation doses between two groups. Group based trajectory modeling was used to determine the two trajectory groups [15]. The trajectory groups were created according to the three measurements of TGF-β1 or PDGF in each patient as a continuous outcome measure and the groups represent clusters of individuals with similar trajectories and outcomes over time [16]. Models were fitted by using the flexmix package [17] of the statistical program R, version 3.3.0, from the R Foundation for Statistical Computing [18]. The relative goodness of fit was assessed using Bayesian information criteria (BIC). Fischer’s exact test was used to test for differences in categorical variables in the two trajectory groups. Linear regression was used to test multivariable associations with the change in GLS over 3 years. IBM SPSS Statistics software, version 25 for Windows (Armonk, NY, USA) was used for statistical testing. *P*-values under 0.05 were considered statistically significant.

## Results

### TGF-β1, PDGF and proBNP

Including all 63 patients, the median TGF-β1 decreased significantly during RT, *p* = 0.003 (Table 1). At the three-year follow-up, the median TGF-β1 level remained lower than before RT, *p* = 0.001. The median PDGF also decreased during RT, *p* = 0.001. The difference in median PDGF levels between before RT and at 3 years remained significant, *p* = 0.046. The median proBNP (*n* = 62) was stable during RT, *p* = 0.325, but increased by the three-year follow-up, *p* < 0.001.

**Table 1:** TGF-β1, PDGF and proBNP levels in the whole study population

		Before RT		After RT		Three years				
	n	Md	IQR	Md	IQR	Md	IQR	p^1^	p^2^	p^3^
TGF-β1 (ng/ml)	63	19.2	(17.1–22.3)	18.8	(14.5–22.0)	17.2	(13.7–21.2)	**0.003**	**0.001**	0.101
PDGF (ng/ml)	63	15.4	(12.6–19.1)	13.8	(11.7–16.2)	15.6	(10.4–18.4)	**0.001**	**0.046**	0.661
proBNP (ng/l)	62	75.5	(42.5–125.5)	75.0	(35.5–132.8)	101.0	(55.5–183.3)	0.325	**< 0.001**	**< 0.001**

*TGF-β1* Transforming growth factor beta 1, *PDGF* Platelet-derived growth factor, *proBNP* N-terminal pro-brain natriuretic peptide, *RT* Radiotherapy, *Md* Median, *IQR* Interquartile range, *p*^*1*^ Change from before to after RT, *p*^*2*^ Change from before to 3 years after RT, *p*^*3*^ Change from after RT to 3 years after RT

The correlations of TGF-β1 and PDGF at corresponding time points and the changes between these time points are shown in Table 2. There were significant correlations between the TGF-β1 and PDGF as well as between the TGF-β1 and proBNP levels (Table 2), but PDGF and proBNP did not correlate together.

**Table 2:** Correlations between TGF-β1, PDGF and proBNP

	TGF-β1 before RT	TGF-β1 after RT	TGF-β1 3 years	Change in TGF-β1 before RT–after RT	Change in TGF-β1 before RT–3 years	Change in TGF-β1 after RT–3 years
PDGF before RT	**0.667**	0.222	0.132	**─0.405**	**─0.480**	─0.088
PDGF after RT	**0.453**	**0.713**	**0.316**	**0.249**	─0.141	**─0.361**
PDGF 3 years	**0.299**	**0.338**	**0.749**	0.022	**0.336**	**0.414**
Change in PDGF before RT–after RT	─0.221	**0.481**	0.246	**0.646**	**0.363**	─0.204
Change in PDGF before RT–3 years	─**0.254**	─0.128	**0.625**	**0.308**	**0.739**	**0.513**
Change in PDGF after RT–3 years	─0.132	**─0.267**	**0.544**	─0.152	**0.600**	**0.826**
proBNP before RT	**0.298**	─0.037	─0.040	─**0.325**	─**0.280**	─0.050
proBNP after RT	**0.265**	─0.041	─0.006	─**0.252**	─0.183	─0.005
proBNP 3 years	**0.293**	─0.060	─0.061	─**0.321**	─**0.297**	─0.070
Change in proBNP before RT–after RT	─0.040	─0.016	0.054	0.101	0.150	0.075
Change in proBNP before RT–3 years	0.114	0.087	0.004	─0.023	─0.111	─0.129
Change in proBNP after RT–3 years	0.212	0.018	0.004	─**0.259**	─0.232	─0.060

*TGF-β1* transforming growth factor beta 1, *PDGF* Platelet-derived growth factor, *proBNP* N-terminal pro-brain natriuretic peptide, *RT* Radiotherapy, Correlation coefficients (Spearman’s rho) in bold are statistically significant (*p* < 0.05)

### TGF-β1, PDGF and baseline characteristics

Age inversely correlated with the TGF-β1 level at 3 years (rho = − 0.310, *p* = 0.013) and the change in TGF-β1 from before RT to 3 years (rho = − 0.280, *p* = 0.026). There was no significant correlation between TGF-β1 and body mass index (BMI) or time from surgery to RT. Furthermore, there was no statistically significant difference in the TGF-β1 levels before RT in groups with different comorbidities or the use of medication, e.g. hypertension, hypothyrosis, coronary artery disease (CAD), smoking, diabetes and use of aromatase inhibitors (AI), tamoxifen, angiotensin convertase inhibitors (ACE), acetylsalicylic acid (ASA), or statins.

PDGF at 3 years also inversely correlated with age (rho = − 0.348, *p* = 0.005), but not with BMI or time from surgery to RT. Statin-users had lower median PDGF levels before RT than did nonusers, 12.0 (10.2–16.5) ng/ml and 16.5 (13.4–20.4) ng/ml, respectively (*p* = 0.024). Furthermore, tamoxifen-users had lower median PDGF levels after RT than nonusers did, 12.1 (11.6–17.3) ng/ml and 15.6 (12.8–20.1) ng/ml, respectively (*p* = 0.041). There were no differences in the median PDGF levels according to the other baseline characteristics.

### TGF-β1 and PDGF levels and echocardiographic measurements

The TGF-β1 and PDGF levels before RT, after RT, at 3 years, and the changes between these time points correlated with different structural and functional parameters in echocardiography. Echocardiographic measurements are presented in Additional file 1: Table S1. The significant correlations for TGF-β1 and PDGF after RT and at 3 years and echocardiography are presented in Additional file 2: Table S2.

#### Correlations with structural echocardiographic measurement

The interventricular septum (IVS) (rho 0.256, *p* = 0.042) and posterior wall (PW) (rho = 0.318, *p* = 0.011) thicknesses and the left ventricular end systolic diameter (LVESD) (rho 0.300, *p* = 0.017) after RT correlated with TGF-β1 before RT. Additionally, the IVS (rho 0.428, *p* < 0.001) and PW (rho = 0.389, *p* = 0.002) thicknesses at 3 years correlated with the TGF-β1 level before RT.

Furthermore, the change in TGF-β1 during RT inversely correlated with the change in septal calibrated integrated backscatter (scIBS) during RT (rho = ─0.289, *p* = 0.023). The change in TGF-β1 from before RT to 3 years inversely correlated with the change in IVS from before RT to 3 years (rho = ─0.255, *p* = 0.044) and the IVS at 3 years (rho = ─0.383, *p* = 0.002).

The PDGF level before RT correlated with the change in IVS from before RT to 3 years (rho = 0.306, *p* = 0.015). The PDGF change during RT correlated inversely with the posterior calibrated integrated backscatter (pcIBS) before RT (rho = ─0.341, *p* = 0.007) and positively with the change in pcIBS during RT (rho = 0.307, *p* = 0.016) and the change in pcIBS from baseline to 3 years (rho = 0.336, *p* = 0.009). The PDGF change from before RT to 3 years correlated positively with the change in left ventricular end diastolic diameter (LVEDD) (rho = 0.267, *p* = 0.035) and negatively with the change in IVS (rho = ─0.385, *p* = 0.002) from before RT to 3 years.

#### Correlations with systolic echocardiographic measurements

The global longitudinal strain (GLS) at 3 years correlated positively with the TGF-β1 level before RT (rho 0.441, *p* < 0.001) and inversely with the TGF-β1 change during RT (rho = ─0.302, *p* = 0.018). Furthermore, the GLS change from before RT to 3 years inversely correlated with the PDGF level before RT (rho = ─0.288, *p* = 0.022). Likewise, the PDGF change during RT correlated with the TAPSE change from before RT to 3 years (rho = 0.262, *p* = 0.045).

#### Correlations with measurements of filling pressure and diastology in echocardiography

The TGF-β1 level before RT inversely correlated with the mitral early inflow wave velocity (mitral E) (rho ─0.300, *p* = 0.017) after RT. The PDGF level before RT inversely correlated with the mitral E before RT (rho = ─0.288, *p* = 0.022), after RT (rho = ─0.416, *p* = 0.001) and at three years (rho = ─0.270, *p* = 0.033).

### TGF-β1 trajectories

Trajectory analysis was performed to group the patients by behavior of TGF-β1. Group 1 (*n* = 19) had significantly higher levels of TGF-β1 before RT, after RT and at 3 years than group 2 (*n* = 44), *p* < 0.001 for all time points (Table 3). In group 1, there was a tendency for TGF-β1 levels to decrease from before to after RT and to the three-year follow-up, *p* = 0.066 and *p* = 0.080 respectively. In group 2, there was a significant decrease in the TGF-β1 level from baseline to after RT, *p* = 0.023, and to the three-year follow-up, *p* = 0.006. The groups were similar in baseline characteristics (Table 3), and there were no significant differences in the proBNP levels at the three time points between the groups.

**Table 3:** TGF-β1 levels and baseline characteristics according to two groups determined by a TGF-β1 trajectory analysis

	n	Group 1	n	Group 2	*p*
TGF-β1 before RT (ng/ml) Md (IQR)	19	25.6 (22.3–28.6)	44	17.8 (15.9–19.9)	**< 0.001**
TGF-β1 after RT (ng/ml), Md (IQR)	19	23.9 (21.3–25.2)	44	16.3 (13.7–19.1)	**< 0.001**
TGF-β1 at 3 years (ng/ml), Md (IQR)	19	22.4 (18.8–25.8)	44	15.0 (12.1–18.8)	**< 0.001**
Age, Md (IQR)	19	64 (58–66)	44	64 (59–68)	0.752
BMI, Md (IQR)	19	26.9 (24.8–32.3)	44	25.7 (24.1–28.7)	0.110
Left-sided BC, n (%)	19	14 (73.7)	44	36 (81.8)	0.508
AI-use, n (%)	19	6 (31.2)	44	15 (34.1)	1.000
Tamoxifen use, n (%)	19	2 (10.5)	44	4 (9.1)	1.000
ACE or ARB use, n (%)	19	5 (26.3)	44	12 (27.3)	1.000
ASA use, n (%)	19	1 (5.3)	44	4 (9.1)	1.000
Beta-blocker use, n (%)	19	4 (21.1)	44	7 (15.9)	0.721
Statin use, n (%)	19	3 (15.8)	44	10 (22.7)	0.738
CAD, n (%)	19	2 (10.5)	44	1 (2.3)	0.214
Diabetes, n (%)	17	3 (17.6)	42	2 (4.8)	0.138
Hypertension, n (%)	19	9 (47.4)	44	15 (34.1)	0.400
Hypothyroidism, n (%)	19	3 (15.8)	44	7 (15.9)	1.000
Smoking, n (%)	19	2 (10.5)	44	5 (11.4)	1.000

*TGF-β1* Transforming growth factor beta 1, *RT* Radiotherapy, *Md* Median, *IQR* Interquartile range, *BMI* Body mass index, *BC* Breast cancer, *AI* Aromatase inhibitor, *ACE* Angiotensin converting enzyme inhibitor, *ARB* Angiotensin II receptor blocker, *ASA* Low dose acetylsalicylic acid, *CAD* Coronary artery disease, *Diabetes* Use of diabetes medication

Echocardiographic parameters by the two trajectory groups are shown in Table 4. Baseline measurements were similar between the two groups. The IVS at 3 years, the PW after RT and the PW at 3 years were significantly different between the groups, *p* = 0.016, *p* = 0.039 and *p* = 0.010, respectively. There was a tendency for a difference in GLS at 3 years, *p* = 0.081. During RT, there was a significant change for group 1 in IVS (*p* = 0.036), PW (*p* = 0.030), TAPSE (*p* = 0.021) and scIBS (*p* = 0.030). For group 2 there was a significant change during RT in TAPSE (*p* = 0.030) and scIBS (*p* = 0.007). During the three-year follow-up, GLS and scIBS significantly worsened from baseline in group 1, *p* = 0.013 and *p* < 0.001, respectively. In group 2, PW decreased and scIBS increased from baseline to 3 years, *p* = 0.028 and *p* = 0.002, respectively. Radiation doses to the heart, left ventricle (LV), right ventricle (RV) and the left anterior descending artery (LAD) were similar between the two trajectory groups (Additional file 3: Table S3).

**Table 4:** Echocardiographic measurements according to TGF-β1 trajectory groups

		Group 1 (*n* = 19)								Group 2 (*n* = 44)								
		Before RT		After RT		3 year				Before RT			After RT		3 year			
	n	Md	(IQR)	Md	(IQR)	Md	(IQR)	p^1^	p^2^	n	Md	(IQR)	Md	(IQR)	Md	(IQR)	p^1^	p^2^
LV measurements																		
LVEDD (mm)	19	46.0	(44.0–48.0)	45.0	(44.0–50.0)	46.0	(42.0–50.0)	0.513	0.446	44	46.0	(44.0–47.0)	46.0	(43.3–47.0)	45.0	(43.0–47.0)	0.904	0.654
LVESD (mm)	19	31.0	(29.0–34.0)	32.0	(28.0–34.0)	31.0	(29.0–35.0)	0.854	0.954	44	31.0	(29.0–32.8)	30.5	(29.0–31.8)	30.0	(29.0–32.0)	0.781	0.896
IVS (mm)	19	10.0	(9.0–11.0)	11.0	(10.0–12.0)	11.0	(10.0–11.0)	**0.036**	0.157	44	10.0	(9.0–11.0)	10.0	(9.0–11.0)	10.0	(9.0–11.0)	0.378	0.775
PW (mm)	19	10.0	(9.0–11.0)	11.0	(10.0–12.0)	9.0	(10.0–11.0)	**0.030**	0.434	44	10.0	(9.0–11.0)	10.0	(9.3–11.0)	9.0	(8.3–10.0)	0.144	**0.028**
LV systolic function																		
LV EF (%)	19	62.0	(56.0–65.0	61.0	(58.0–65.0)	60.0	(57.0–63.0)	0.886	0.144	44	62.0	(60.0–65.0	63	(59.3–65.0)	61.5	(57.0–63.0)	1.000	0.092
GLS (%)	17	─17.0	(─20.5-─16.0)	─17.0	(─19.5-─15.5)	─16.0	(─18.5-─14.0)	0.373	**0.006**	44	─18.0	(─19.8-─15.0)	─17.0	(─20.0-─15.0)	─18.0	(─20.0-─16.0)	0.159	0.762
LV diastolic function																		
Mitral inflow E (cm/s)	19	73.5	(66.2–81.9)	66.1	(57.2–79.0)	66.2	(61.7–78.4)	0.113	0.106	44	75.4	(63.8–87.3)	72.4	(60.3–80.0)	71.4	(62.0–89.0)	0.139	0.242
Ee’ ratio	19	8.3	(6.8–10.2)	7.8	(7.1–11.1)	8.7	(7.1–10.2)	0.701	0.347	44	9.5	(7.7–11.4)	9.3	(7.1–10.2)	8.9	(7.7–11.0)	0.076	0.337
RV function																		
TAPSE (mm)	17	24.0	(21.0–28.0)	21.0	(18.5–24.5)	22.0	(20.5–27.5)	**0.021**	0.363	42	24.5	(20.8–27.3)	22.0	(19.0–26.0)	24.0	(20.0–26.0)	**0.001**	0.119
TR gradient (mmHg)	12	21.0	(18.3–26.0)	20.5	(17.3–24.0)	23.5	(19.3–28.5)	0.351	0.381	35	21.0	(17.0–25.0)	22.0	(19.0–24.0)	24.0	(20.0–29.0	0.811	**< 0.001**
Tissue characterization																		
scIBS (dB)	17	15.5	(12.5–21.3)	19.5	(13.7–25.1)	23.3	(20.1–25.8)	**0.031**	**< 0.001**	44	17.6	(14.1–22.1)	19.6	(16.3–24.2)	20.3	(18.8–22.8)	**0.007**	**0.002**
rcIBS (dB)	17	18.7	(14.8–21.8)	22.9	(20.1–25.9)	23.5	(19.1–27.0)	**0.011**	**0.013**	44	21.1	(17.1–24.6)	23.0	(18.0–26.9)	24.1	(20.3–27.0)	0.208	**0.021**
pcIBS (dB)	17	10.3	(7.6–11.9)	9.2	(8.2–12.1)	9.4	(6.5–13.1)	0.579	0.854	43	9.8	(6.8–13.9)	10.3	(8.3–12.5)	10.5	(6.8–13.4)	0.888	0.959

*TGF-β1* Transforming growth factor beta 1, *RT* Radiotherapy, *Md* Median, *IQR* Interquartile range, *p*^*1*^ p-value for before to after RT, *p*^*2*^ p-value for before to 3 years after RT, *LV* Left ventricle, *LVEDD* Left ventricle end diastolic diameter, *LVESD* Left ventricle end systolic diameter, *IVS* Interventricular septum thickness, *PW* Posterior wall thickness, *EF* Ejection fraction, *GLS* Global longitudinal strain, *Mitral inflow E* First peak of diastole, *Ee’* Pulsed tissue doppler e’ velocity, *RV* Right ventricle, *TAPSE* Tricuspid annular plane systolic excursion, *TR gradient* Tricuspid regurgitation maximal gradient, *scIBS* Septal calibrated integrated backscatter, *rcIBS* Right ventricle integrated backscatter, *pcIBS* Posterior wall of left ventricle integrated backscatter

To further explore the association between TGF-β1 and GLS suggested by correlation and the significant worsening in trajectory group 1, multivariable linear regression analysis was performed. In the model, TGF-β1 trajectory group 1 (β = 0.27, p = 0.013), left-sided breast cancer (β = 0.39, *p* = 0.001) and the use of AI (β = 0.29, *p* = 0.011) were significantly associated with a reduction in GLS from before RT to 3 years. Additionally, there was tendency for age to be associated (β = 0.18, *p* = 0.071) with worsening GLS during the three-year follow-up. These factors explained 33% of the change in GLS.

### PDGF trajectories

A trajectory analysis was also performed for PDGF. PDGF levels were significantly higher at all time-points in group 1 (*n* = 8) than in group 2 (*n* = 55), *p* < 0.001 (Additional file 4: Table S4) for all time-points. The groups did not differ in baseline characteristics (Additional file 4: Table S4). The change in PDGF was only significant in group 2 from before to after RT, *p* = 0.001.

Only scIBS at 3 years was significantly higher in group 1 than group 2, *p* = 0.044. The elevated PDGF levels in group 1 were not associated with more changes in echocardiographic parameters, but the group 1 was too small for a meaningful comparison (Additional file 5: Table S5). Furthermore, radiation doses to the heart, LV, RV or LAD were similar in the groups (Additional file 4: Table S4).

## Discussion

### Elevated baseline TGF-β1 associates with echocardiographic changes

The most important finding in our study was the association of elevated TGF-β1 before RT with a decline in LV systolic function, namely, impairment in GLS during the three-year follow-up. This association was apparent in the correlation between TGF-β1 and GLS at 3 years and further with the trajectory analysis in which patients were grouped into two groups according to TGF-β1 behavior. Group 1 had significantly higher baseline TGF-β1 levels than group 2. At baseline, the echocardiographic parameters were similar, but RT induced a thickening of the IVS and PW during RT in group 1, but not in group 2. These changes most likely depict RT-induced inflammatory changes. During the three-year follow-up, group 1 had a significant worsening of GLS and group 2 did not. In multivariable analysis, the trajectory group 1 remained a significant factor in predicting GLS worsening during the 3 years in addition to AI use and left-sided breast cancer, which we have reported in earlier to affect echocardiographic parameters [12, 13]. Left-sided breast cancer associates with higher radiation doses to the heart [19], which probably explains the significance of the side. Radiation dose is a significant factor determining cardiovascular risk of breast cancer patients and dose-volume constraints are in clinical use to decrease this risk [20]. Radiation doses were similar between the groups, as the grouping mostly reflects a difference in baseline TGF-β1. The worsening in GLS is probably the most clinically significant echocardiographic change, as GLS is an excellent early predictor of major adverse cardiac events [21]. Additionally, the correlation between the levels of TGF-β1 and proBNP, an accepted marker of heart failure [22], at all three time-points further supports the association of elevated TGF-β1 levels and cardiovascular changes.

In previous literature, two other studies with adjuvant external beam RT [8] and intracavitary partial breast brachytherapy [9] reported elevated circulating baseline TGF-β1 levels in patients who developed radiation-induced fibrosis of the breast. Studies with genetic mutations also support the idea that baseline TGF-β1 levels may predispose individuals to normal tissue toxicity from RT. Circulating levels of TGF-β1 are, at least partially, controlled genetically [3]. The TGF-β1 C-509 T variant allele (rs1800469) is associated with elevated levels of circulating TGF-β1 [3]. Prospective studies present contradictory evidence of the association between TGF-β1 C-509 T variant allele and radiation-induced fibrosis of the breast [4, 5]. There is some evidence that mutations in TGF-β1 genes are linked to cardiovascular risk. In patients receiving postoperative RT for breast cancer, patients with the TGF-β1 29C > T variant allele, associated with low TGF-β1 levels, had an increased cardiovascular risk with HR 1.79. However, in this study, there was no association between CV mortality and radiation dose [7]. Furthermore, the role of TGF-β1 is not clear in cardiovascular diseases. The evidence on the role of TGF-β1 in atherosclerosis is contradictory, but most studies suggest TGF-β1 inhibits atherosclerosis [23]. In contrast, elevated levels of TGF-β1 are associated with, for example, hypertrophic cardiomyopathy [23].

### Changes in TGF-β1 levels

Earlier, we reported that TGF-β1 and PDGF decrease during RT [10], but this study shows that TGF-β1 remains at a lower level at 3 years compared to before RT. The decreases were small but statistically significant. In a suggested model, RT is thought to induce a cytokine release [2], but since we only have measurements for before and after RT, a cytokine release could have taken place earlier during the RT course. One previous study with intraoperative RT (IORT) for breast cancer reported that IORT had no effect on the TGF-β1 levels measured from wound fluid [24]. We found that a decrease in TGF-β1 during RT and the three-year follow-up correlated with worsening LV systolic function e.g. GLS. Additionally, the three-year change was correlated with an increased echodensity of the myocardium, scIBS, and LV measurements. The inverse correlations between the change in TGF-β1 levels and proBNP levels, further suggest that the decrease in TGF-β1 may be associated with an increased cardiovascular risk.

### PDGF levels and associations with echocardiographic measurements

We also found associations between elevated baseline PDGF levels and LV systolic function, e.g. worsening GLS, and LV diastolic function, e.g. decreased mitral E. The change in PDGF during RT also predicted impairment in GLS over 3 years. The trajectory analysis did not further support the usability of PDGF in predicting RT-induced echocardiographic changes. Additionally, there were no correlations between PDGF and proBNP. Therefore, despite the strong correlation between the two biomarkers, TGF-β1 seems to be more usable than PDGF in predicting the cardiovascular effects of RT. To our knowledge, no previous studies exist about the PDGF and RT-induced toxicity in humans.

### Limitations

Although we now present results with 3 years of follow-up, the follow-up time is still short, considering that the increased risk of cardiovascular effects of RT takes years to manifest. Longer follow-up will show whether the echocardiographic changes lead to clinical cardiovascular morbidity. This may require larger studies than ours. Additionally, the underlying cause of elevation of TGF-β1 is not known nor do we have information on how well other risk factors of cardiovascular diseases, such as elevated blood pressure, diabetes, and hyperlipidemia, are controlled in the patients. These factors could influence the results.

## Conclusions

Our finding supports that elevated TGF-β1 before RT is a risk factor for the susceptibility to normal tissue toxicity and is associated with RT-induced changes in echocardiography, namely, impairment in GLS, a measure of LV systolic function. An elevated TGF-β1 level is an independent risk factor for the impairment in GLS in addition to AI use and left-sided breast cancer, which is likely due to higher radiation doses in left-sided breast cancer. Furthermore, a decrease in TGF-β1 during the three-year follow-up also correlated with echocardiographic changes. More studies and longer follow-up are needed to confirm whether elevated TGF-β1 can be used to determine which patients are at an increased risk for radiation-induced heart disease. Further, this information could even be useful when designing future cardioprotective trials.

## Additional files

Additional file 1: **Table S1.** Echocardiographic measurements in the whole study population. (DOCX 33 kb)

Additional file 2: **Table S2.** Echocardiographic measurements with significant correlations with TGF-β1 and PDGF after RT and at 3 years. (DOCX 30 kb)

Additional file 3: **Table S3.** Radiation doses to the heart according to groups determined by TGF-β1 trajectory analysis. (DOCX 31 kb)

Additional file 4: **Table S4.** PDGF levels and baseline characteristics according to groups determined by PDGF trajectory analysis. (DOCX 33 kb)

Additional file 5: **Table S5.** Echocardiographic measurements according to PDGF trajectory groups. (DOCX 38 kb)

## Data Availability

The datasets used and analyzed during the current study are available from the corresponding author on reasonable request.

## Abbreviations

ACE: Angiotensin converting enzyme inhibitor
AI: Aromatase inhibitor
ARB: Angiotensin II receptor blocker
ASA: Low dose acetylsalicylic acid
BMI: Body mass index
CAD: Coronary artery disease
DCIS: Ductal carcinoma in situ
Ee’: Pulsed tissue doppler e’ velocity
EF: Ejection fraction
GLS: Global longitudinal strain
IQR: Interquartile range
IVS: Interventricular septum thickness
LV: Left ventricle
LVEDD: Left ventricle end diastolic diameter
LVESD: Left ventricle end systolic diameter
Md: Median
Mitral inflow E: First peak of diastole
pcIBS: Posterior wall of left ventricle integrated backscatter
PDGF: Platelet-derived growth factor
proBNP: N-terminal pro-brain natriuretic peptide
PTV: Planning target volume
PW: Posterior wall thickness
rcIBS: Right ventricle integrated backscatter
RT: Radiotherapy
RV: Right ventricle
scIBS: Septal calibrated integrated backscatter
TAPSE: Tricuspid annular plane systolic excursion
TGF-β1: Transforming growth factor beta 1
TR gradient: Tricuspid regurgitation maximal gradient
