# Supplementary material for: Transforming growth factor beta 1 levels predict echocardiographic changes at three years after adjuvant radiotherapy for breast cancer
Source: Radiat Oncol. 2019 Aug 30;14:155. doi: 10.1186/s13014-019-1366-1 (PMC6717329; doi:10.1186/s13014-019-1366-1)
Supplement: Supplementary file 1 — Table S1. Echocardiographic measurements in the whole study population. (DOCX 33 kb) [file 13014_2019_1366_MOESM1_ESM.docx]

**Table S1** Echocardiographic measurements in the whole study population.

|  |  | Before RT | | After RT | | 3 years | |  |  |  |
| --- | --- | --- | --- | --- | --- | --- | --- | --- | --- | --- |
|  | n | Md | (IQR) | Md | (IQR) | Md | (IQR) | p¹ | p² | p³ |
| **LV measurements** | | |  |  |  |  |  |  |  |  |
| LVEDD (mm) | 63 | 46.0 | (44.0-47.0) | 46.0 | (44.0-48.0) | 45.0 | (42.0-48.0) | 0.716 | 0.413 | 0.982 |
| LVESD (mm) | 63 | 31.0 | (29.0-33.0) | 31.0 | (29.0-33.0) | 31.0 | (29.0-32.0) | 0.680 | 0.889 | 0.897 |
| IVS (mm) | 63 | 10.0 | (9.0-11.0) | 10.0 | (9.0-11.0) | 10.0 | (9.0-11.0) | **0.034** | 0.231 | 0.598 |
| PW (mm) | 63 | 10.0 | (9.0-11.0) | 10.0 | (10.0-11.0) | 9.0 | (9.0-11.0) | **0.011** | 0.175 | **<0.001** |
| **LV systolic function** | | |  |  |  |  |  |  |  |  |
| LV EF (%) | 63 | 62.0 | (60.0-65.0) | 63.0 | (59.0-65.0) | 61.0 | (57.0-63.0) | 0.936 | **0.027** | **0.007** |
| GLS (%) | 61 | ─18.0 | (─20.0-─16.0) | ─17.0 | (─20.0-─15.0) | ─17.0 | (─19.0-─16.0) | 0.106 | 0.103 | 0.693 |
| **LV diastolic function** | | |  |  |  |  |  |  |  |  |
| Mitral inflow E (cm/s) | 63 | 75.0 | (64.1-84.8) | 68.1 | (60.2-79.5) | 69.1 | (61.9-85.3) | **0.026** | 0.062 | 0.842 |
| Ee’ ratio | 63 | 9.3 | (7.4-11.3) | 8.5 | (7.1-10.3) | 8.8 | (7.5-10.9) | 0.218 | 0.818 | 0.111 |
| **RV function** | | |  |  |  |  |  |  |  |  |
| TAPSE (mm) | 59 | 24.0 | (21.0-28.0) | 22.0 | (19.0-25.0) | 23.0 | (20.0-26.0) | **<0.001** | 0.060 | 0.110 |
| TR gradient (mmHg) | 47 | 21.0 | (18.0-25.0) | 22.0 | (18.0-24.0) | 24.0 | (20.0-29.0) | 0.493 | **0.001** | **0.001** |
| **Tissue characterization** | | |  |  |  |  |  |  |  |  |
| scIBS (dB) | 61 | 17.2 | (13.9-21.5) | 19.5 | (15.8-24.4) | 21.1 | (19.0-23.6) | **<0.001** | **<0.001** | **0.029** |
| rcIBS (dB) | 61 | 20.6 | (17.1-24.2) | 22.9 | (18.8-26.8) | 24.0 | (20.2-26.9) | **0.014** | **0.001** | 0.210 |
| pcIBS (dB) | 60 | 10.0 | (6.9-13.5) | 9.6 | (8.3-12.5) | 10.4 | (6.7-13.4) | 0.862 | 0.961 | 0.744 |

RT, radiotherapy; Md, median; IQR, interquartile range; p¹, p-value for before to after RT; p², p-value for before to 3 years after RT; p³, p-value for after RT to 3 years after RT; LV, left ventricle; LVEDD, left ventricle end diastolic diameter; LVESD, left ventricle end systolic diameter; IVS, interventricular septum thickness; PW, posterior wall thickness; EF, ejection fraction; GLS, global longitudinal strain; Mitral inflow E, first peak of diastole; Ee’, pulsed tissue doppler e’ velocity; RV, right ventricle; TAPSE, tricuspid annular plane systolic excursion; TR gradient, tricuspid regurgitation maximal gradient; scIBS, septal calibrated integrated backscatter; rcIBS, right ventricle integrated backscatter; pcIBS, posterior wall of left ventricle integrated backscatter
