# Supplementary material for: Transforming growth factor beta 1 levels predict echocardiographic changes at three years after adjuvant radiotherapy for breast cancer
Source: Radiat Oncol. 2019 Aug 30;14:155. doi: 10.1186/s13014-019-1366-1 (PMC6717329; doi:10.1186/s13014-019-1366-1)
Supplement: Supplementary file 2 — Table S2. Echocardiographic measurements with significant correlations with TGF-β1 and PDGF after RT and at 3 years. (DOCX 30 kb) [file 13014_2019_1366_MOESM2_ESM.docx]

**Table S2** Echocardiographic measurements with significant correlations with TGF-β1 and PDGF after RT and at three years.

|  | TGF–β1  after RT | TGF–β1  three years | PDGF  after RT | PDGF  three years |
| --- | --- | --- | --- | --- |
| **Systolic function** | |  |  |  |
| TAPSE before RT | –0.047 | –0.227 | –0.190 | **–0.294** |
| LV EF at three years | –0.012 | –0.189 | –0.156 | **–0.269** |
| **Filling pressure and diastolic function** | |  |  |  |
| Ee’ ratio before RT | 0.055 | –0.204 | –0.174 | **–0.282** |
| Mitral E after RT | 0.010 | –0.026 | **–0.273** | **–0.269** |
| Change in Tr gradient from before to after RT | **–0.310** | –0.031 | –0.282 | 0.036 |
| Change in Mitral E from before RT to three years | **–0.265** | 0.036 | –0.043 | 0.037 |

TGF-β1, transforming growth factor beta 1; PDGF, platelet-derived growth factor; RT, radiotherapy; TAPSE, tricuspid annular plane systolic excursion; LV EF, left ventricular ejection fraction; Ee’, pulsed tissue doppler e’ velocity ; Mitral E, mitral early inflow wave velocity; Tr gradient, tricuspid gradient
