# Supplementary material for: Transforming growth factor beta 1 levels predict echocardiographic changes at three years after adjuvant radiotherapy for breast cancer
Source: Radiat Oncol. 2019 Aug 30;14:155. doi: 10.1186/s13014-019-1366-1 (PMC6717329; doi:10.1186/s13014-019-1366-1)
Supplement: Supplementary file 3 — Table S3. Radiation doses to the heart according to groups determined by TGF-β1 trajectory analysis. (DOCX 31 kb) [file 13014_2019_1366_MOESM3_ESM.docx]

**Table S3** Radiation doses to the heart according to groups determined by TGF-β1 trajectory analysis.

|  | Group 1 (n=24) | | Group 2 (n=47) | |  |
| --- | --- | --- | --- | --- | --- |
|  | Md | (IQR) | Md | (IQR) | p |
| Mean heart dose (Gy) | 3.0 | (1.0-4.2) | 1.8 | (1.0-3.6) | 0.222 |
| V20 Gy to heart (%) | 3.4 | (0-5.3) | 1.4 | (0-4.8) | 0.376 |
| Mean LV dose (Gy) | 4.0 | (1.1-7.0) | 2.7 | (1.3-5.5) | 0.379 |
| V20 Gy to LV (%) | 3.6 | (0-9.6) | 1.8 | (0-7.2) | 0.502 |
| Mean RV dose (Gy) | 1.9 | (0.9-2.7) | 1.5 | (1.0-2.9) | 0.561 |
| V20 Gy to RV (%) | 0 | (0-0.7) | 0 | (0-1.4) | 0.923 |
| Mean LAD dose (Gy) | 20.0 | (1.1-31.7) | 10.1 | (2.0-24.2) | 0.164 |
| V20 Gy to LAD (%) | 45.8 | (0-73.8) | 19.5 | (0-48.9) | 0.128 |

TGF-β1, transforming growth factor beta 1; Md, median; IQR, interquartile range; V20 Gy, percentage of structure volume receiving 20 Gy; LV, left ventricle; RV, right ventricle; LAD, left anterior descending coronary artery.
