# Supplementary material for: Transforming growth factor beta 1 levels predict echocardiographic changes at three years after adjuvant radiotherapy for breast cancer
Source: Radiat Oncol. 2019 Aug 30;14:155. doi: 10.1186/s13014-019-1366-1 (PMC6717329; doi:10.1186/s13014-019-1366-1)
Supplement: Supplementary file 4 — Table S4. PDGF levels and baseline characteristics according to groups determined by PDGF trajectory analysis. (DOCX 33 kb) [file 13014_2019_1366_MOESM4_ESM.docx]

**Table S4** PDGF levels and baseline characteristics according to groups determined by PDGF trajectory analysis.

|  | Group 1 | | | Group 2 | | |  |
| --- | --- | --- | --- | --- | --- | --- | --- |
|  | n |  | | n |  | | p |
| PDGF before RT (ng/ml), Md (IQR) | 8 | 28.9 | (25.7-35.7) | 55 | 14.6 | (12.1-17.4) | **<0.001** |
| PDGF after RT (ng/ml), Md (IQR) | 8 | 25.6 | (22.2-36.3) | 55 | 13.4 | (11.1-15.8) | **<0.001** |
| PDGF at 3 years (ng/ml), Md (IQR) | 8 | 25.5 | (20.6-37.0) | 55 | 14.6 | (9.5-17.4) | **<0.001** |
| Age, Md (IQR) | 8 | 63.0 | (55.3-65.5) | 55 | 64.0 | (58.0-67.0) | 0.378 |
| BMI, Md (IQR) | 8 | 25.7 | (22.3-28.4) | 55 | 26.2 | (24.2-30.2) | 0.333 |
| Left-sided BC, n (%) | 8 | 7 | (87.5) | 55 | 43 | (78.2) | 1.000 |
| AI-use, n (%) | 8 | 1 | (12.5) | 55 | 20 | (36.4) | 0.250 |
| Tamoxifen use, n (%) | 8 | 0 | (0.0) | 55 | 6 | (10.9) | 1.000 |
| ACE or ARB use, n (%) | 8 | 0 | (0.0) | 55 | 17 | (30.9) | 0.095 |
| ASA use, n (%) | 8 | 0 | (0.0) | 55 | 5 | (9.1) | 1.000 |
| Beta-blocker use, n (%) | 8 | 0 | (0.0) | 55 | 11 | (20.0) | 0.331 |
| Statin use, n (%) | 8 | 1 | (12.5) | 55 | 12 | (21.8) | 1.000 |
| CAD, n (%) | 8 | 0 | (0.0) | 55 | 3 | (5.5) | 1.000 |
| Diabetes, n (%), | 8 | 0 | (0.0) | 46 | 5 | (9.8) | 1.000 |
| Hypertension, n (%) | 8 | 1 | (12.5) | 55 | 23 | (41.8) | 0.141 |
| Hypothyroidism, n (%) | 8 | 1 | (12.5) | 55 | 9 | (16.4) | 1.000 |
| Smoking, n (%) | 8 | 1 | (12.5) | 55 | 6 | (10.9) | 1.000 |

PDGF, platelet-derived growth factor; RT, radiotherapy; RT, radiotherapy; Md, median; IQR, interquartile range; BMI, body mass index; BC, breast cancer; AI, aromatase inhibitor; ACE, angiotensin converting enzyme inhibitor; ARB, angiotensin II receptor blocker; ASA, low dose acetylsalicylic acid; CAD, coronary artery disease; Diabetes, use of diabetes medication
