# Supplementary material for: Transforming growth factor beta 1 levels predict echocardiographic changes at three years after adjuvant radiotherapy for breast cancer
Source: Radiat Oncol. 2019 Aug 30;14:155. doi: 10.1186/s13014-019-1366-1 (PMC6717329; doi:10.1186/s13014-019-1366-1)
Supplement: Supplementary file 5 — Table S5. Echocardiographic measurements according to PDGF trajectory groups. (DOCX 38 kb) [file 13014_2019_1366_MOESM5_ESM.docx]

**Table S5** Echocardiographic measurements according to PDGF trajectory groups.

|  |  | Group 1 | | | | | | | | | |  | Group 2 | | | | | | | |  |  |  |  |  |  |
| --- | --- | --- | --- | --- | --- | --- | --- | --- | --- | --- | --- | --- | --- | --- | --- | --- | --- | --- | --- | --- | --- | --- | --- | --- | --- | --- |
|  |  | Before RT | | After RT | | | 3 year | | |  | |  | Before RT | | | After RT | | 3 year | | |  |  | |  |  |  |
|  | n | Md | (IQR) | | Md | (IQR) | | Md | (IQR) | p1 | p2 | | n | Md | (IQR) | Md | (IQR) | | Md | (IQR) | p^1^ | p^2^ | |  |  |  |
| **LV measurements** | | | | |  |  | |  |  |  |  | |  |  |  |  |  | |  |  |  |  | |  |  |  |
| LVEDD (mm) | 8 | 46.5 | (44.3-47.0 | | 44.0 | (44.0-48.5) | | 45.0 | (42.5-50.5) | 0.469 | 0.563 | | 55 | 46.0 | (44.0-48.0) | 46.0 | (43.0-48.0) | | 45.0 | (42.0-48.0) | 0.978 | 0.534 | |  |  |  |
| LVESD (mm) | 8 | 30.5 | (29.3-33.5) | | 31.0 | (24.0-34.5) | | 30.0 | (28.3-34.8) | 0.234 | 0.563 | | 55 | 31.0 | (29.0-33.0) | 31.0 | (29.0-32.0) | | 31.0 | (29.0-33.0) | 0.841 | 0.932 | |  |  |  |
| IVS (mm) | 8 | 10.0 | (8.3-10.8) | | 10.5 | (9.3-11.0) | | 10.0 | (9.3-11.0) | 0.125 | 0.344 | | 55 | 10.0 | (9.0-11.0) | 10.0 | (9.0-11.0) | | 10.0 | (9.0-11.0) | 0.105 | 0.418 | |  |  |  |
| PW (mm) | 8 | 10.0 | (9.0-11.0) | | 10.5 | (10.0-11.8) | | 9.5 | (8.3-10.8) | 0.070 | 0.781 | | 55 | 10.0 | (9.0-11.0) | 10.0 | (10.0-11.0) | | 9.0 | (9.0-11.0) | **0.047** | 0.209 | |  |  |  |
| **LV systolic function** | | | | |  |  | |  |  |  |  | |  |  |  |  |  | |  |  |  |  | |  |  |  |
| LV EF (%) | 8 | 62.5 | (55.3-65.8) | | 61.5 | (58.0-64.0) | | 59.0 | (53.5-62.5) | 0.906 | 0.344 | | 55 | 62.0 | (60.0-65.0) | 63.0 | (59.0-65.0) | | 61.0 | (57.0-63.0) | 0.850 | **0.064** | |  |  |  |
| GLS (%) | 8 | ─19.0 | (─21.0-─16.3) | | ─18.5 | (─20.5-─15.5) | | ─18.0 | (─21.5-─14.0) | 0.359 | 0.313 | | 53 | ─18.0 | (─20.0-─15.0) | ─17.0 | (─20.0-─15.0) | | ─17.0 | (─19.0-─16.0) | 0.188 | 0.203 | |  |  |  |
| **LV diastolic function** | | | | |  |  | |  |  |  |  | |  |  |  |  |  | |  |  |  |  | |  |  |  |
| Mitral inflow E (cm/s) | 8 | 75.1 | (64.0-84.6) | | 62.0 | (53.2-75.2) | | 65.3 | (51.6-87.5) | **0.039** | 0.625 | | 55 | 75.0 | (64.1-84.8) | 69.1 | (60.5-80.1) | | 69.6 | (60.5-80.1) | 0.129 | 0.109 | |  |  |  |
| Ee’ ratio | 8 | 8.2 | (6.2-9.5) | | 8.9 | (7.2-11.0) | | 8.5 | (7.3-10.8) | 0.672 | 0.437 | | 55 | 9.4 | (7.4-11.4) | 8.5 | (7.1-10.2) | | 8.9 | (7.6-11.0) | 0.125 | 0.573 | |  |  |  |
| **RV function** | | | | |  |  | |  |  |  |  | |  |  |  |  |  | |  |  |  |  | |  |  |  |
| TAPSE (mm) | 6 | 23.0 | (20.8-28.8) | | 20.5 | (17.8-23.5) | | 22.0 | (19.0-27.0) | 0.188 | 0.438 | | 53 | 24.0 | (21.0-27.5) | 22.0 | (19.0-25.5) | | 24.0 | (20.0-26.0) | **<0.001** | 0.093 | |  |  |  |
| TR gradient (mmHg) | 6 | 20.0 | (14.8-24.8) | | 19.5 | (15.0-23.3) | | 22.0 | (20.0-25.0) | 0.563 | 0.563 | | 41 | 21.0 | (18.5-25.5) | 22.0 | (18.5-24.5) | | 25.0 | (19.0-29.0) | 0.712 | **0.001** | |  |  |  |
| **Tissue characterization** | | | | |  |  | |  |  |  |  | |  |  |  |  |  | |  |  |  |  | |  |  |  |
| scIBS (dB) | 8 | 15.5 | (12.2-20.6) | | 21.5 | (13.4-24.2) | | 16.7 | (11.9-23.2) | **0.016** | **0.023** | | 53 | 17.7 | (14.0-21.5) | 19.2 | (15.8-24.5) | | 21.1 | (19.2-23.6) | **0.007** | **<0.001** | |  |  |  |
| rcIBS (dB) | 8 | 20.2 | (17.4-22.3) | | 23.1 | (18.6-24.5) | | 23.0 | (20.9-26.1) | 0.383 | 0.383 | | 53 | 20.6 | (17.0-24.3) | 22.9 | (18.8-27.1) | | 24.2 | (20.0-27.1) | **0.043** | **0.003** | |  |  |  |
| pcIBS (dB) | 8 | 9.5 | (6.5-10.9) | | 10.3 | (8.6-14.7) | | 7.6 | (6.3-11.7) | 0.313 | 0.844 | | 52 | 10.0 | (6.9-13.7) | 9.6 | (7.9-12.3) | | 10.8 | (6.7-13.5) | 0.733 | 0.877 | |  |  |  |

PDGF, platelet-derived growth factor; RT, radiotherapy; Md, median; IQR, interquartile range; p¹, p-value for before to after RT; p², p-value for before to 3 years after RT; p³, p-value for after RT to 3 years after RT; LV, left ventricle; LVEDD, left ventricle end diastolic diameter; LVESD, left ventricle end systolic diameter; IVS, interventricular septum thickness; PW, posterior wall thickness; EF, ejection fraction; GLS, global longitudinal strain; Mitral inflow E, first peak of diastole; Ee’, pulsed tissue doppler e’ velocity ; RV, right ventricle; TAPSE, tricuspid annular plane systolic excursion; TR gradient, tricuspid regurgitation maximal gradient; scIBS, septal calibrated integrated backscatter; rcIBS, right ventricle integrated backscatter; pcIBS, posterior wall of left ventricle integrated backscatter
